# A Bayesian unified framework for risk estimation and cluster identification in small area health data analysis

**DOI:** 10.1371/journal.pone.0231935

**Published:** 2020-05-07

**Authors:** K. C. Flórez, A. Corberán-Vallet, A. Iftimi, J. D. Bermúdez

**Affiliations:** Department of Statistics and Operations Research, University of Valencia, Valencia, Spain; University of Bonn, Bonn-Aachen International Center for IT, GERMANY

## Abstract

Many statistical models have been proposed to analyse small area disease data with the aim of describing spatial variation in disease risk. In this paper, we propose a Bayesian hierarchical model that simultaneously allows for risk estimation and cluster identification. Our model formulation assumes that there is an unknown number of risk classes and small areas are assigned to a risk class by means of independent allocation variables. Therefore, areas within each cluster are assumed to share a common risk but they may be geographically separated. The posterior distribution of the parameter representing the number of risk classes is estimated using a novel procedure that combines its prior distribution with an efficient estimate of the marginal likelihood of the data given this parameter. An extension of the model incorporating covariates is also shown. These covariates may incorporate additional information on the problem or they may account for spatial correlation in the data. We illustrate the performance of the proposed model through both a simulation study and a case study of reported cases of varicella in the city of Valencia, Spain.

## Introduction

In the last decades there has been an increasing interest in the area of disease mapping, with the consequent development of numerous statistical techniques for the analysis of public health data. Health data usually consist of aggregated counts of disease within administrative units (small areas) such as zip codes, municipalities, etc. The objective of disease mapping is then to investigate geographic disease variation across the predefined study region.

It is usually assumed that disease counts are described by a discrete probability model. For relatively rare diseases, we can assume a Poisson likelihood with a mean which is a function of the expected counts of disease and the area-specific relative risks. The expected counts of disease represent the background population effect and they are usually estimated using a standard population rate. Once estimated, they are assumed fixed and known and the emphasis is placed on the study of the unknown relative risks, which measure the local deviation of the disease. For finite populations, we could consider a Binomial distribution instead ([1], chapter 5).

A wide range of statistical models have been developed to provide suitable relative risk estimates. By borrowing information across the areas, these models provide smoothed risk surfaces and improve local estimates. The most common approach to relative risk modeling is to assume a logarithm link to a linear predictor which is a function of spatial random effects and possible covariate effects. For instance, the convolution model decomposes the log of the relative risk as a function of spatially correlated and uncorrelated random effects [2]. This formulation has been found to be a robust and an appropriate model to describe disease variation and it is widely used in practice [3, 4]. On some occasions, a spatial trend model capturing large-scale variation of risk over the study region may provide an accurate description of the data ([1], chapter 5). Distance-risk models can be used when a specific point source is suspected to be responsible for an increased disease risk [5]. An alternative model-based approach for smoothing risks in a spatial context is based on the use of splines. Recently, penalized splines and area-specific random effects have been combined to model large-scale spatial trend together with individual variation [6, 7]. Goicoa et al. [8] provide a comparison of CAR and P-spline models in terms of smoothing and detection of high risk areas.

Identification of areas of significantly elevated risk also plays an important role in public health, since it facilitates the implementation of targeted public health interventions. One of the most widely used tests for cluster detection is the spatial scan statistic [9] and its extensions (see, for instance, [10]). Often, it is convenient to consider clusters as a residual feature of the data, and so different residual diagnostics have been proposed to identify extreme regions ([1], chapter 6). The posterior probability that the relative risk exceeds a reference threshold has also been used to classify areas as high risk areas [11, 12]. A major concern with the usefulness of these measures is that they are sensitive to the model chosen. Besides, they attempt to detect clusters from a spatially smoothed risk surface, which may be problematic.

Models that explicitly describe the clustering behaviour of the data have also been designed. Some recent studies address risk estimation and cluster identification simultaneously. In [13] the authors propose a non-parametric mixture model within an empirical Bayes context to identify population heterogeneity. Knorr-Held and Raßer [14] propose a Bayesian partition model where small areas are combined in clusters attending a distance measure that ensures that clusters are connected. Denison and Holmes [15] present a similar Bayesian model to estimate the risk surface. The main difference is that clusters are defined using the Voronoi tessellation. A related partition model is proposed in [16], where the authors introduce a class of Markov connected component field priors to incorporate some prior information about clusters. However, the model is not intended to provide a flexible modeling of the risk surface. Hidden Markov models have also been proposed in a disease mapping context to analyze spatial heterogeneity of disease count data. For instance, [17] present a class of hidden Markov random field (HMRF) models related to an underlying finite-mixture model for the Poisson rates. To allow for spatial correlation, the allocation variables are modeled through a Potts model. One feature of this model is that disconnected areas can have the same label. An alternative frequentist approach for direct classified risk mapping based on a discrete HMRF model can be found in [18]. A different approach has been recently proposed in [19]. In that paper, the authors propose a two-step methodology where a spatially adjusted hierarchical agglomerative clustering algorithm is applied to data prior to the study period to elicit potential cluster structures. Then, for each cluster configuration, a Bayesian hierarchical model is fitted to the study data and the final cluster structure is chosen based on the deviance information criterion. A Bayesian spatio-temporal model can be found in [20].

In this paper, we develop a flexible parametric Bayesian hierarchical model that simultaneously allows for risk estimation and cluster detection in a purely spatial context. We assume that there is an unknown number *k* of risk levels that describe the risk surface and small area count data are assigned to a risk class by means of independent and identically distributed Multinomial indicator vectors. Therefore, geographically separated small areas with a similar risk can be grouped in the same class. It is important to emphasize that, unlike previous model formulations, we do not impose any spatial correlation, which allows us to explore different risk structures. Conditioning on *k*, we develop an MCMC algorithm to sample from the joint posterior distribution of the model parameters. A novel procedure is then developed to estimate the value of *k*. In particular, it is obtained from its posterior distribution, which is computed as a function of the marginal likelihood of the data given *k* and its prior distribution. The proposed model can be straightforwardly extended to incorporate information from covariates. These covariates can account for spatial correlation in the data if present.

We organize this paper as follows. In Section 2 we present the model formulation and describe our MCMC implementation. In Section 3 we analyze the performance of the model using a simulation study. Section 4 provides an application to reported varicella cases in the city of Valencia, Spain. We conclude with a general discussion of the proposed methodology and provide directions for future research.

## Model formulation and Bayesian analysis

Let us assume that the study region is divided in *m* contiguous non-overlapping small areas, and let *y*_*i*_ represent the count of disease observed in the *i*-th area, *i* = 1, 2, …, *m*. We also assume that there is a piecewise constant risk surface that describes geographic disease variation, and so counts of disease are modeled with the following mixture of Poisson distributions:

$$f\left(y_{i}|k,\eta ,p,e_{i}\right)=\sum_{j=1}^{k}\text{Po}\left(y_{i}|e_{i}\eta_{j}\right)p_{j},$$ (1)

where *e*_*i*_ is the expected count of disease, *k* is the number of risk classes (that can be viewed as clusters), *η* = (*η*_1_, *η*_2_, …, *η*_*k*_)′ is the vector of risks, and *p* = (*p*_1_, *p*_2_, …, *p*_*k*_)′ is the vector of probabilities associated with the risk classes, with *p*_*j*_ ≥ 0 and $\sum_{j=1}^{k}p_{j}=1$. If the small areas in the study region have a similar disease risk, then the components of the risk vector *η* will take similar values. It may even be the case that only one latent risk (*k* = 1) suffices to describe counts of disease across the entire study region. On the other hand, a risk surface exhibiting abrupt jumps will dominate.

Let *z*_*i*_ = (*z*_*i*1_, *z*_*i*2_, …, *z*_*ik*_)′ be the latent indicator vector that assigns the small area *i* to one of the *k* risk classes; that is, *z*_*ij*_ is equal to one if the relative risk for area *i* corresponds to *η*_*j*_ and zero otherwise. Eq (1) can then be formulated as:

$$\begin{array}{ccc} y_{i}|k,z_{i},\eta ,e_{i} & ∼ & \text{Po}(y_{i}|e_{i}z_{i}^{\prime }\eta ),\\ z_{i}|p & ∼ & \text{Mult}(z_{i}|n=1,p). \end{array}$$ (2)

Since a common prior distribution is assumed for all the indicator vectors *z*_*i*_, *i* = 1, 2, …, *m*, this model formulation implies that the prior probabilities of the areas belonging to every possible class are the same.

### Bayesian analysis of the model when *k* is known

Let us suppose first that the number of risk classes *k* is known. Prior distributions for the model parameters *η* and *p* can be specified as follows. In order to mitigate the label switching problem common in mixture models, we propose to order the risk classes. This can be easily achieved by using the transformation:

$$\eta_{j}^{*}=\frac{\eta_{j}}{1+\eta_{j}},\;j=1,\ldots ,k,$$

which transforms the interval [0, +∞) into [0, 1], and introducing a vector of cut-off points *v* = (*v*_0_ = 0, *v*_1_, …, *v*_*k*−1_, *v*_*k*_ = 1)′ so that:

$$f\left(\eta^{*}|v\right)=\prod_{j=1}^{k}\text{Un}\left(v_{j-1},v_{j}\right)=\prod_{j=1}^{k}\frac{1}{v_{j}-v_{j-1}}I_{\left(v_{j-1},v_{j}\right)}\left(\eta_{j}^{*}\right),$$

where I_*A*(.)_ denotes the indicator function of the set *A*. The prior distribution for the risk vector *η* is then given by:

$$f\left(\eta |v\right)=\prod_{j=1}^{k}\frac{1}{\left(v_{j}-v_{j-1}\right)}\frac{1}{{\left(1+\eta_{j}\right)}^{2}}I_{\left(\frac{v_{j-1}}{1-v_{j-1}},\frac{v_{j}}{1-v_{j}}\right)}\left(\eta_{j}\right).$$ (3)

The prior distribution that we propose for the vector *v* is based on the distances between a cut-off point and the following, that is *d*_*j*_ = *v*_*j*_ − *v*_*j*−1_. Note that these distances are positive and add up to one, so a natural prior distribution for the vector *d* = (*d*_1_, *d*_2_, …, *d*_*k*_)′ is the Dirichlet distribution, *d* ∼ *Dir*(*d*|*γ*). We assign *γ* a particular value, specifically *γ*_*j*_ = 1 ∀*j*, which corresponds to the flat Dirichlet distribution. Under that assumption, *E*(*d*_*j*_) = 1/*k*. However, other values of *γ* are also possible. The prior distribution for the vector *v* is then given by:

$$f\left(v\right)=\prod_{j=1}^{k}{\left(v_{j}-v_{j-1}\right)}^{\gamma_{j-1}},$$ (4)

if *v*_0_ = 0 ≤ *v*_1_ ≤ … ≤ *v*_*k*−1_ ≤ *v*_*k*_ = 1.

As a prior distribution for *p* we consider the Dirichlet distribution:

p∼Dir(p|α), (5)

where *α* is also assigned a particular value. The choice of *α* here is important, since it may affect the posterior results. Values larger than 1 may prevent some classes from being empty (see, for instance, [21] and the references therein). Under the assumption that the number of risk classes *k* is known, we can use a weakly informative prior, such as *α*_*j*_ = 2 ∀*j*, which facilitates the estimation of the risks within each class and, consequently, convergence of MCMC chains. Other values of *α* are also possible (for more details, see the sensitivity analysis performed in the case study section).

The joint posterior distribution for the model parameters is given by:

$$\begin{array}{ccc} f(z,p,\eta ,v|y,k,e) & \propto & f(y|k,z,\eta ,e)f(z|p)f(p)f(\eta |v)f(v)\\ & \propto & \left[\prod_{i=1}^{m}{\left(z_{i}^{\prime }\eta \right)}^{y_{i}}\text{exp}\left\{-e_{i}z_{i}^{\prime }\eta \right\}][\prod_{i=1}^{m}z_{i}^{\prime }p][\prod_{j=1}^{k}p_{j}^{\alpha_{j}-1}\right]\\ & & \prod_{j=1}^{k}\frac{1}{\left(v_{j}-v_{j-1}\right)}\frac{1}{{\left(1+\eta_{j}\right)}^{2}}I_{\left(\frac{v_{j-1}}{1-v_{j-1}},\frac{v_{j}}{1-v_{j}}\right)}\left(\eta_{j}\right)\\ & & \prod_{j=1}^{k}{\left(v_{j}-v_{j-1}\right)}^{\gamma_{j}-1}I_{\left(v_{j-1},v_{j+1}\right)}\left(v_{j}\right). \end{array}$$ (6)

This posterior distribution is analytically intractable but can be sampled using MCMC simulation techniques.

#### MCMC algorithm

To sample from the joint posterior distribution of the model parameters, we suggest an MCMC simulation algorithm based on the Gibbs sampling procedure, which requires the full conditional posterior distributions to be known [22].

The latent indicator vector *z*_*i*_ ∈ {*E*_1_, *E*_2_, …, *E*_*k*_}, *E*_*j*_ being the *j*-th column of the identity matrix *I*_*k*_. The probability of each value is given by:

$$\begin{array}{ccc} f(z_{i}=E_{j}|y,k,p,\eta ,v,e) & \propto & f\left(y_{i}|k,z_{i}=E_{j},\eta ,e_{i}\right)f\left(z_{i}=E_{j}|p\right)\\ & \propto & \eta_{j}^{y_{i}}\text{exp}\left\{-e_{i}\eta_{j}\right\}p_{j}. \end{array}$$

So, the full conditional posterior distribution for each vector *z*_*i*_, *i* = 1, 2, …, *m*, is the discrete distribution defined as:

$$f\left(z_{i}=E_{j}|y,k,p,\eta ,v,e\right)=\frac{\eta_{j}^{y_{i}}\text{exp}\left\{-e_{i}\eta_{j}\right\}p_{j}}{\sum_{l=1}^{k}\left(\eta_{l}^{y_{i}}\text{exp}\left\{-e_{i}\eta_{l}\right\}p_{l}\right)},\;j=1,\ldots ,k.$$ (7)

The full conditional posterior distribution for parameter *p* is given by:

$$\begin{array}{ccc} f(p|y,k,z,\eta ,v,e) & \propto & f(z|p)f(p)\\ & \propto & \prod_{j=1}^{k}p_{j}^{\left(\sum_{i=1}^{m}z_{ij}\right)+\alpha_{j}-1}; \end{array}$$ (8)

that is, *p* ∼ *Dir*(*p*|*α* + *z*_+._), where *z*_+._ is the resultant vector after adding the columns of the indicator matrix *z*.

The full conditional posterior distribution of the latent risk *η*_*j*_, *j* = 1, 2, …, *k*, can be obtained as:

$$\begin{array}{ccc} f(\eta_{j}|y,k,z,p,\eta_{-j},v,e) & \propto & \prod_{i:z_{i}=E_{j}}f\left(y_{i}|k,z_{i}=E_{j},\eta_{j},e_{i}\right)f\left(\eta_{j}|v\right)\\ & \propto & {\left(\eta_{j}\right)}^{y_{\left(j\right)}}\text{exp}\left\{-e_{\left(j\right)}\eta_{j}\right\}\;\frac{1}{{\left(1+\eta_{j}\right)}^{2}}\;I_{\left(\frac{v_{j-1}}{1-v_{j-1}},\frac{v_{j}}{1-v_{j}}\right)}\left(\eta_{j}\right)\\ & \propto & \text{Ga}\left[\eta_{j}|y_{\left(j\right)}+1,e_{\left(j\right)}\right]\;\frac{1}{{\left(1+\eta_{j}\right)}^{2}}\;I_{\left(\frac{v_{j-1}}{1-v_{j-1}},\frac{v_{j}}{1-v_{j}}\right)}\left(\eta_{j}\right), \end{array}$$ (9)

where *y*_(*j*)_ and *e*_(*j*)_ represent, respectively, the sum of the observed and expected counts of disease of those areas that are assigned to the *j*-th risk class. Note that if the *j*-th risk class is empty, the posterior distribution of parameter *η*_*j*_ corresponds to the prior distribution.

Finally, the full conditional posterior distribution of each cut-off point *v*_*j*_, *j* = 1, 2, …, *k* − 1, is given by:

$$\begin{array}{ccc} f(v_{j}|y,k,z,p,\eta ,v_{-j},e) & \propto & f(\eta |v)f(v)\\ & \propto & \frac{1}{v_{j}-v_{j-1}}I_{\left(\frac{v_{j-1}}{1-v_{j-1}},\frac{v_{j}}{1-v_{j}}\right)}\left(\eta_{j}\right)\\ & & \frac{1}{v_{j+1}-v_{j}}I_{\left(\frac{v_{j}}{1-v_{j}},\frac{v_{j+1}}{1-v_{j+1}}\right)}\left(\eta_{j+1}\right)\\ & & {\left(v_{j}-v_{j-1}\right)}^{\gamma_{j}-1}{\left(v_{j+1}-v_{j}\right)}^{\gamma_{j+1}-1}I_{\left(v_{j-1},v_{j+1}\right)}\left(v_{j}\right)\\ & \propto & {\left(v_{j}-v_{j-1}\right)}^{\gamma_{j}-2}{\left(v_{j+1}-v_{j}\right)}^{\gamma_{j+1}-2}I_{\left(\frac{\eta_{j}}{1+\eta_{j}},\frac{\eta_{j+1}}{1+\eta_{j+1}}\right)}\left(v_{j}\right). \end{array}$$ (10)

Note that only the last indicator function is necessary, since the previous ones imply $v_{j-1}<\frac{\eta_{j}}{1+\eta_{j}}<v_{j}$ and $v_{j}<\frac{\eta_{j+1}}{1+\eta_{j+1}}<v_{j+1}$.

Details on how to simulate from these full conditional posterior distributions are given in S1 Appendix.

### Bayesian analysis of the model when *k* is unknown

In practice, however, the number of risk classes is unknown and so it has to be estimated. Previous approaches to estimation of *k* include the use of a reversible jump MCMC algorithm [14, 17], randomized model search strategies [16] or a penalty based approach that fixes *k* to be overly large and penalises values in the extreme risk classes [20]. In the context of Bayesian finite mixture models, [21] propose a criterion to determine the appropriate number of latent classes based on the posterior distribution of the class proportions. Concretely, the mixture model is first estimated with a relatively large number of latent classes. The true number of latent classes is then estimated as the posterior mode of the number of non-empty classes (where a class is defined as empty in an MCMC iteration if the proportion of observations assigned to that class is below a certain cut-off). This criterion, which can be easily computed using standard software for Bayesian analysis, has proved to perform well if the hyperparameters of the Dirichlet prior on the class proportions is sufficiently vague.

Here, we pursue a novel alternative methodology, which provides a discrete posterior distribution *f*(*k*|*y*, *e*) on the integers {1, 2, …, *K*}. Our alternative is based on the marginal likelihood of the data given the parameter *k*:

f(y|k,e)=∫∫f(y|k,p,η,e)f(p,η|k)dpdη. (11)

Note that this marginal likelihood can be approximated by Monte-Carlo integration using a sample ${\left\{p^{\left(n\right)},\eta^{\left(n\right)}\right\}}_{n=1}^{N}$ from the prior distribution of parameters *p* and *η*. However, this approach may not be efficient if the prior distribution is non-informative. In order to reduce the Monte-Carlo error, we define the following procedure. Let us first assume that *k* = 1. In that case, the only parameter of the model is *η*, and the marginal likelihood can be formulated as:

$$\begin{array}{c} f\left(y|k=1,e\right)=\frac{\prod_{i=1}^{m}e_{i}^{y_{i}}}{\prod_{i=1}^{m}y_{i}!}\int_{0}^{\infty }\eta^{\sum_{i=1}^{m}y_{i}}\text{exp}\left\{-\eta \sum_{i=1}^{m}e_{i}\right\}\frac{1}{{\left(1+\eta \right)}^{2}}d\eta \\ \;=\frac{\prod_{i=1}^{m}e_{i}^{y_{i}}}{\prod_{i=1}^{m}y_{i}!}\Gamma (1+\sum_{i=1}^{m}y_{i}){\left(\sum_{i=1}^{m}e_{i}\right)}^{-(1+\sum_{i=1}^{m}y_{i})}\int_{0}^{\infty }\frac{1}{{\left(1+\eta \right)}^{2}}\text{Ga}(\eta |1+\sum_{i=1}^{m}y_{i},\sum_{i=1}^{m}e_{i})d\eta \\ \;=\frac{\prod_{i=1}^{m}e_{i}^{y_{i}}}{\prod_{i=1}^{m}y_{i}!}\Gamma (1+\sum_{i=1}^{m}y_{i}){\left(\sum_{i=1}^{m}e_{i}\right)}^{-(1+\sum_{i=1}^{m}y_{i})}E\left({\left(1+\eta \right)}^{-2}\right). \end{array}$$ (12)

Given a sample ${\left\{\eta^{\left(n\right)}\right\}}_{n=1}^{N}$ from $\text{Ga}(\eta |1+\sum_{i=1}^{m}y_{i},\sum_{i=1}^{m}e_{i})$, Eq (12) can be estimated as:

$$\hat{f}\left(y|k=1,e\right)=\frac{\prod_{i=1}^{m}e_{i}^{y_{i}}}{\prod_{i=1}^{m}y_{i}!}\Gamma (1+\sum_{i=1}^{m}y_{i}){\left(\sum_{i=1}^{m}e_{i}\right)}^{-(1+\sum_{i=1}^{m}y_{i})}\frac{1}{N}\sum_{n=1}^{N}\frac{1}{{\left(1+\eta^{\left(n\right)}\right)}^{2}}\;.$$

In the general case *k* > 1, *g*(*η*, *p*, *d*|*y*, *k*, *e*) = *g*(*η*|*y*, *k*, *d*, *e*)*g*(*p*|*y*, *k*, *d*, *e*)*g*(*d*|*k*, *e*) is defined as a naive approximation to the posterior distribution of the model parameters and it is used as an importance function. We next describe this importance function when *k* = 2. Its generalization for values of *k* > 2 is straightforward. If *k* = 2, *η* = (*η*_1_, *η*_2_)′, *p* = (*p*_1_, 1 − *p*_1_)′, and *d* = (*d*_1_, 1 − *d*_1_)′. In particular, we assume that *g*(*d*_1_|*k*) = Be(*d*_1_|*γ*_1_, *γ*_2_). Once the value of *d*_1_ has been observed, each small area is assigned to the first risk class if $\frac{y_{i}}{e_{i}}<\frac{d_{1}}{1-d_{1}}$. Let *m*_1_ be the number of small areas assigned to the first risk class, and *m*_2_ = *m* − *m*_1_ the number of small areas assigned to the second risk class. Function *g*(*p*_1_|*y*, *k*, *d*, *e*) is defined as Be(*p*_1_|*α*_1_+ *m*_1_, *α*_2_+ *m*_2_). Finally, *g*(*η*_1_|*y*, *k*, *d*, *e*) and *g*(*η*_2_|*y*, *k*, *d*, *e*) are defined, respectively, from the empirical distribution of ${\left\{y_{i}|e_{i}\right\}}_{i:y_{i}/e_{i}<d_{1}/(1-d_{1})}$ and ${\left\{y_{i}|e_{i}\right\}}_{i:y_{i}/e_{i}>d_{1}/(1-d_{1})}$. The marginal likelihood function can then be expressed as:

$$\begin{array}{ccc} f(y|k,e) & = & \int \int \int f(y|k,p,\eta ,d,e)f(p,\eta ,d|k)dpd\eta dd\\ & = & \int \int \int f(y|k,p,\eta ,e)f(p,\eta |k,d)f(d|k)dpd\eta dd\\ & = & \int \int \int f\left(y|k,p,\eta ,e\right)f\left(p,\eta |k,d\right)\frac{g(\eta ,p,d|y,k,e)}{g(\eta ,p|y,k,d,e)}dpd\eta dd\\ & = & E(\frac{f(y|k,p,\eta ,e)f(p,\eta |k,d)}{g(\eta ,p|y,k,d,e)}), \end{array}$$ (13)

which can be easily estimated using a sample from *g*(*η*, *p*, *d*|*y*, *k*, *e*).

Once the marginal likelihood function of the data given the parameter *k* has been estimated for *k* = 1, 2, …, *K*, the value of *k* can be obtained from its posterior distribution:

$$f\left(k|y,e\right)\propto \hat{f}\left(y|k,e\right)f\left(k\right)$$ (14)

where *f*(*k*) = *q*_*k*_ is a discrete prior distribution on the integers {1, 2, …, *K*}.

The advantage of this procedure is that it allows for cluster identification and risk estimation simultaneously. If we are interested in identifying the number of risk classes, we can select the value of *k* within the set {1, 2, …, *K*} with the highest posterior probability. This criterion is similar to the one proposed in [21] in the sense that one value of *k* is selected from the posterior distribution. However, if the final objective of the study is to estimate the relative risk of each small area *i*, *i* = 1, 2, …, *m*, we can derive relative risk estimates accounting for model uncertainty through posterior averaging. That is, given the parameters of the model, the relative risk of area *i* is:

$$\theta_{i}=\sum_{j=1}^{k}\eta_{j}P\left(z_{i}=E_{j}|y,k,p,\eta ,v,e\right)$$ (15)

Using a sample generated from the joint posterior distribution of the model parameters with the Gibbs sampling procedure, the relative risks can be estimated as:

$$\hat{E}\left(\theta_{i}|y,k,e\right)=\frac{1}{N}\sum_{n=1}^{N}\theta_{i}^{\left(n\right)}=\frac{1}{N}\sum_{n=1}^{N}{\left(z_{i}^{\left(n\right)}\right)}^{\prime }\eta^{\left(n\right)}.$$

In the general case of *k* unknown, we can use the posterior distribution of parameter *k* to estimate the relative risk for area *i* as:

$$\hat{E}\left(\theta_{i}|y,e\right)=\sum_{k=1}^{K}\hat{E}\left(\theta_{i}|y,k,e\right)f\left(k|y,e\right).$$ (16)

### Extension of the model with covariates

Let us know assume that we have information from *L* covariates, *x*_*i*_ being the *L* × 1 vector of covariate information corresponding to the *i*-th small area, *i* = 1, 2, …, *m*. In this case, counts of disease are modeled as:

$$\begin{array}{ccc} y_{i}|k,z_{i},\eta ,\beta ,e_{i},x_{i} & ∼ & \text{Po}(y_{i}|e_{i}\cdot \text{exp}\left\{x_{i}^{\prime }\beta \right\}\cdot z_{i}^{\prime }\eta ),\\ z_{i}|p & ∼ & \text{Mult}(z_{i}|n=1,p), \end{array}$$ (17)

where the difference with respect to Eq (2) is the incorporation of the extra term $\text{exp}\{x_{i}^{\prime }\beta \}$ in the mean of the Poisson distribution.

The prior distribution assumed for parameter *β* is the multivariate Gaussian distribution with zero mean vector and covariance matrix Σ. As hyperprior for parameter Σ, we propose an inverse Wishart distribution with *ν* degrees of freedom and scale matrix Ψ. A typically used relatively uninformative inverse Wishart prior sets *ν* = *L* + 1 and Ψ = *I*_*L*_ (see, for instance, [23]). The particular case *L* = 1 corresponds to an inverted Gamma distribution $\sigma_{\beta }^{2}∼Ga^{-1}\left(a,b\right)$ with *a* = *ν*/2 and *b* = Ψ/2.

The full conditional posterior distributions for parameters *z*_*i*_, *i* = 1, 2, …, *m*, *p*, *η*_*j*_, and *v*_*j*_, *j* = 1, 2, …, *k*, remain as those in Eqs (7)–(10), the only difference is that *e*_*i*_ is substituted by $e_{i}\cdot \text{exp}\left\{x_{i}^{\prime }\beta \right\}$.

The full conditional posterior distributions for parameters *β* and Σ are given by:

$$\begin{array}{ccc} f(\beta |y,k,z,p,\eta ,v,\Sigma ,e,x) & \propto & \text{exp}\{{\left(\sum_{i=1}^{m}y_{i}x_{i}\right)}^{\prime }\beta \}\text{exp}\{-\sum_{i=1}^{m}e_{i}\cdot \text{exp}\left\{x_{i}^{\prime }\beta \right\}\cdot z_{i}^{\prime }\eta \}\\ & & \text{exp}\{-\frac{1}{2}\beta^{\prime }\Sigma^{-1}\beta \}. \end{array}$$ (18)

$$\begin{array}{ccc} f(\Sigma |y,k,z,p,\eta ,v,\beta ,e,x) & \propto & {\left|\Sigma \right|}^{-\frac{\nu +L+2}{2}}\text{exp}\{-\frac{1}{2}tr(\left(\beta \beta^{\prime }+\Psi \right)\Sigma^{-1})\}. \end{array}$$ (19)

Simulation from the full conditional posterior distribution of parameter *β* can be done using the Metropolis algorithm. In particular, we propose to use the uniform distribution in a ball in $R^{L}$ centered at the previous simulated value of *β* and radius *r* as the proposal density. The full conditional posterior distribution of parameter Σ is an inverse Wishart distribution with *ν*+ 1 degrees of freedom and scale matrix *ββ*′ + Ψ. Simulation from this distribution is straightforward.

As explained in the introduction, most of the statistical models that have been proposed to explain the spatial pattern in small area disease data incorporate spatially autocorrelated random effects, which account for any spatial autocorrelation remaining in the data after the known covariate effects have been accounted for. Because many covariates vary spatially, spatial confounding between spatially structured random effects and fixed effects is a well-known problem. One possible solution is to use a multi-stage modelling process (see, for instance, [24]). It is important to emphasize here that, because the proposed model does not impose any spatial correlation, it is not affected by collinearity problems, and so fixed-effects can be properly estimated.

## Simulation study

We present here some of the results obtained in an extensive simulation study that we conducted to assess the effectiveness of the proposed model to recover the true underlying risk surface (for more details, see [25]). All the analysis was performed using the free statistical software R.

### Data

We used the city of Valencia (Spain), which consists of *m* = 86 contiguous boroughs, as the base map to generate the observed disease count data at borough level. To calculate the expected counts of disease, we used the population size of each borough and assumed an incidence rate *r* = 30 per 10^4^ persons. In *Scenario* 1, the parameters assumed for the simulation were *k* = 3, *η* = (0.5, 1.0, 3.5), and *p* = (0.2, 0.3, 0.5). In *Scenario* 2, the parameters assumed for the simulation were *k* = 7, *η* = (0.2, 0.5, 0.8, 1.0, 1.5, 2.5, 3.5), and *p* = (1/7, 1/7, 1/7, 1/7, 1/7, 1/7, 1/7). To generate the observed disease counts within each scenario, we first assigned the small areas to the different risk levels simulating from a discrete distribution with probabilities given by the corresponding vector *p*. Then, each *y*_*i*_, *i* = 1, 2, …, *m* = 86, was simulated from a Poisson distribution with mean $e_{i}z_{i}^{\prime }\eta$, where *e*_*i*_ was calculated taking into account the population size of the *i*-th borough and the assumed value of *r*, and $z_{i}^{\prime }\eta$ is the risk corresponding to the risk class where the *i*-th borough was assigned.

For comparative purposes, we also simulated data from the convolution model [2]. In *Scenario* 3, the relative risk for the *i*-th borough (*θ*_*i*_) was simulated as:

$$\theta_{i}=\text{exp}\left\{\rho +u_{i}+v_{i}\right\}$$ (20)

where $\rho ∼N(0,\sigma_{\rho }^{2})$ is the overall level of the relative risk in the study region; *u* = (*u*_1_, *u*_2_, …, *u*_*m*_)′ represents the spatially correlated random effect, following a CAR model with variance $\sigma_{u}^{2}$; and *v* = (*v*_1_, *v*_2_, …, *v*_*m*_)′ is assumed to be a realization of a multivariate Gaussian distribution with zero mean vector and covariance matrix $\sigma_{v}^{2}I_{m}$. The values of the standard deviances were (*σ*_*ρ*_, *σ*_*u*_, *σ*_*v*_) = (0.01, 0.05, 0.1). The observed disease count for the *i*-th borough was then simulated from a Poisson distribution with mean *e*_*i*_ *θ*_*i*_, where *e*_*i*_ was calculated taking into account the population size of the borough and an incidence rate *r* = 30 per 10^4^ persons.

Finally, we also considered a scenario where different risks were assigned to each borough without imposing any spatial correlation between risks in nearby areas. In particular, in *Scenario* 4, the relative risk for the *i*-th borough was simulated as:

$$\theta_{i}=\text{exp}\left\{\rho +v_{i}\right\}$$ (21)

where parameters *ρ* and *v*_*i*_ are defined as those in Eq (20). The values of the standard deviances were (*σ*_*ρ*_, *σ*_*v*_) = (0.01, 0.1). This scenario represents a situation where the relative risks are not spatially correlated but, unlike our model formulation, the small areas are not assigned to risk classes.

To allow for sampling variability, we simulated 100 data sets for each scenario. The results presented are averaged over these 100 realizations.

### Evaluation measures

We mainly used two measures to evaluate the performance of our model: The root mean squared error (RMSE) and the percentage of correspondence.

Let *θ*_*i*_ and ${\hat{\theta }}_{i}$ be the true and the estimated relative risk for the *i*-th small area, *i* = 1, 2, …, *m*. The RMSE is defined as $\sqrt{\frac{1}{m}\sum_{i=1}^{m}{\left(\theta_{i}-\hat{\theta_{i}}\right)}^{2}}$.

In the analysis of each data set with our model, we first assumed that the value of *k* was known and simulated 5000 samples from the posterior distribution of the model parameters using the MCMC simulation algorithm described in S1 Appendix. The values of *k* that we considered ranged from *k* = 1 to *k* = 10; that is, we sequentially implemented the MCMC algorithm for *k* = 1, 2, …, *K* = 10. We calculated then the posterior distribution of parameter *k* as previously explained (see Eq (14)) using 100000 samples in the Monte-Carlo integration step. Finally, the estimated relative risk for each borough was calculated using Eq (16). The resulting RMSE is represented by RMSE_mod_. For comparative purposes, we also show the RMSE_smr_ based on the standardized mortality rate (${\hat{\theta }}_{i}=y_{i}/e_{i}$) and the RMSE_BYM_ that was obtained by fitting the convolution model to the simulated dataset. To carry out this analysis, we used the free statistical software WinBUGS.

The percentage of correspondence is calculated as the percentage of data sets within each scenario that satisfy that the value of *k* with the highest posterior probability corresponds to the true value of *k*. Note that this measure can only be calculated for *Scenario* 1 and *Scenario* 2, since the data were simulated from our model and so we know the true value of *k*.

### Results

For *Scenario* 1, the median and interquartile range of the estimated values of *η* and *p* when we assume that *k* is known and equal to 3 are displayed in Table 1. As can be seen, when we condition on the true number of risk classes, the risks within each class are accurately estimated and the proportion of areas assigned to each risk class coincide with the real probabilities of the classes.

**Table 1:** Scenario 1: Median (interquartile range) of the estimated values of *η* and *p* when we assume *k* = 3. True values are *η* = (0.5, 1.0, 3.5) and *p* = (0.2, 0.3, 0.5).

$\hat{\eta_{1}}$	$\hat{\eta_{2}}$	$\hat{\eta_{3}}$
0.49 (0.06)	1.00 (0.06)	3.50 (0.07)
$\hat{p_{1}}$	$\hat{p_{2}}$	$\hat{p_{3}}$
0.22 (0.07)	0.29 (0.07)	0.49 (0.06)

We next consider *k* as an additional parameter of the model and compute its posterior distribution. Table 2 shows the percentage of data sets (out of the 100) that select each possible value of *k* considered (from 1 to 10) as the most probable value. In this scenario, a percentage of correspondence equal to 94% is achieved, being the mean of the posterior probability of the value *k* = 3 equal to 0.79.

**Table 2:** Scenario 1: Percentage of data sets that satisfy that the highest probability of the posterior distribution of *k* coincides with each possible value of *k* considered.

*k*	1	2	3	4	5	6	7	8	9	10
	0%	0%	94%	4%	2%	0%	0%	0%	0%	0%

The corresponding results for *Scenario* 2 are presented in Tables 3 and 4. As in the previous scenario, when we condition on the true number of risk classes (*k* = 7 here), the estimated risks within each class and the probabilities of these classes are close to the real values. As expected, however, the difficulty of identifying the risk classes increases with the number of risk classes used in the simulation and also when there is a low separation between the risks. Under this scenario, the values of *k* that are most often selected are *k* = 7, 8, and 9. In this case, the mean of the posterior probability of the value *k* = 7 is 0.20.

**Table 3:** Scenario 2: Median (interquartile range) of the estimated values of *η* and *p* when we assume *k* = 7. True values are *η* = (0.2, 0.5, 0.8, 1.0, 1.5, 2.5, 3.5) and *p* = (1/7, 1/7, 1/7, 1/7, 1/7, 1/7, 1/7).

$\hat{\eta_{1}}$	$\hat{\eta_{2}}$	$\hat{\eta_{3}}$	$\hat{\eta_{4}}$	$\hat{\eta_{5}}$	$\hat{\eta_{6}}$	$\hat{\eta_{7}}$
0.18 (0.05)	0.46 (0.08)	0.75 (0.08)	1.09 (0.12)	1.65 (0.14)	2.56 (0.18)	4.38 (3.38)
$\hat{p_{1}}$	$\hat{p_{2}}$	$\hat{p_{3}}$	$\hat{p_{4}}$	$\hat{p_{5}}$	$\hat{p_{6}}$	$\hat{p_{7}}$
0.13 (0.03)	0.14 (0.03)	0.16 (0.04)	0.16 (0.04)	0.13 (0.03)	0.14 (0.04)	0.14 (0.05)

**Table 4:** Scenario 2: Percentage of data sets that satisfy that the highest probability of the posterior distribution of *k* coincides with each possible value of *k* considered.

*k*	1	2	3	4	5	6	7	8	9	10
	0%	0%	0%	0%	3%	12%	19%	25%	27%	14%

A comparison with the results provided by the criterion proposed in [21] to select the number of risk classes is shown in S2 Appendix.

The mean of three RMSE previously described are shown in Table 5. This table also displays the 95% Bayesian prediction interval for the difference between the RMSE_BYM_ and the RMSE_mod_ that would be obtained after applying the convolution model and the proposed model to a new data set. As expected, the RMSE_smr_ is the highest one, while the differences between the RMSE_mod_ and the RMSE_BYM_ are not significant except when the data show a marked clustering behaviour. On those occasions, the proposed model provides more satisfactory results. When counts of disease are either spatially correlated or do not show a clustering behaviour, the proposed model is able to capture such behaviour and the relative risk estimates are similar to those provided by the convolution model.

**Table 5:** Mean of the RMSEs obtained in the estimation of the relative risks for the small areas and 95% Bayesian prediction interval for the difference between the RMSE_BYM_ and the RMSE_mod_ that would be obtained after applying the convolution model and the proposed model to a new data set.

	RMSE_smr_	RMSE_mod_	RMSE_BYM_	95% Pred Int
Scenario 1	0.422	0.204	0.397	[0.063,0.322]
Scenario 2	0.342	0.293	0.320	[-0.033,0.088]
Scenario 3	0.286	0.105	0.100	[-0.023,0.012]
Scenario 4	0.284	0.098	0.094	[-0.020,0.011]

## Case study

In this section we study the number of reported varicella cases in the city of Valencia for year 2013. These data were obtained from the Surveillance Service and Epidemiological Control, General Division of Epidemiology and Health Surveillance—Department of Public Health, Generalitat Valenciana. Since the data provided were aggregated counts of disease at borough level, we did not have access to any identifying information of patients.

Varicella, also known as chickenpox, is an acute and highly contagious viral, airborne disease. It is primarily a disease of children, characterized by blister-like rash, itching, tiredness, and fever. It usually resolves by itself within a couple of weeks. Immunity following infection is considered to be long-lasting and reinfections are rare.

The city of Valencia (the third largest city in Spain) consists of 19 districts divided into 87 boroughs. Districts 17, 18 and 19 are thinly populated and far from the urban core. Hence, we consider here the analysis of varicella data in 70 boroughs of Valencia (corresponding to districts 1-16) for year 2013. The number of cases registered in these districts represents over 98% of the total.

In our analysis of the data, we have first assumed that the value of *k* is known and we have simulated 5000 samples from the posterior distribution of the model parameters using the MCMC simulation algorithm described in S1 Appendix. We performed a sensitivity analysis to assess the influence of the values assumed for the hyperparameters on posterior results. We considered values of *γ* and *α* different to the ones proposed in the description of the Bayesian analysis of the model. Concretely, we also assumed *γ*_*j*_ = 2 and *α*_*j*_ = 0.4 or *α*_*j*_ = 1, ∀*j*. In all cases, the correlation among the posterior estimates of the parameters was greater than 0.998. The results presented here correspond to the values *γ*_*j*_ = 1 and *α*_*j*_ = 2. The values of *k* that we have considered range from *k* = 1 to *k* = 10. We have calculated then the posterior distribution of parameter *k* using 100000 samples in the Monte-Carlo integration step. This posterior distribution is shown in Table 6.

**Table 6:** Case study: Posterior distribution of parameter *k*.

*k*	1	2	3	4	5	6	7	8	9	10
	0	0	0.01	0.10	0.40	0.20	0.13	0.08	0.05	0.03

If the objective of the study is to identify clusters of disease, we would select the value of *k* = 5 as the number of risk classes. The posterior estimates of the risks and the probabilities associated with the risk classes are given in Table 7.

**Table 7:** Case study: Median (interquartile range) of the estimated values of *η* and *p* when we assume *k* = 5.

$\hat{\eta_{1}}$	$\hat{\eta_{2}}$	$\hat{\eta_{3}}$	$\hat{\eta_{4}}$	$\hat{\eta_{5}}$
0.46 (0.22)	0.82 (0.26)	1.12 (0.30)	1.85 (0.84)	5.34 (0.62)
$\hat{p_{1}}$	$\hat{p_{2}}$	$\hat{p_{3}}$	$\hat{p_{4}}$	$\hat{p_{5}}$
0.19 (0.19)	0.34 (0.21)	0.26 (0.24)	0.10 (0.12)	0.05 (0.03)

**Fig 1:**
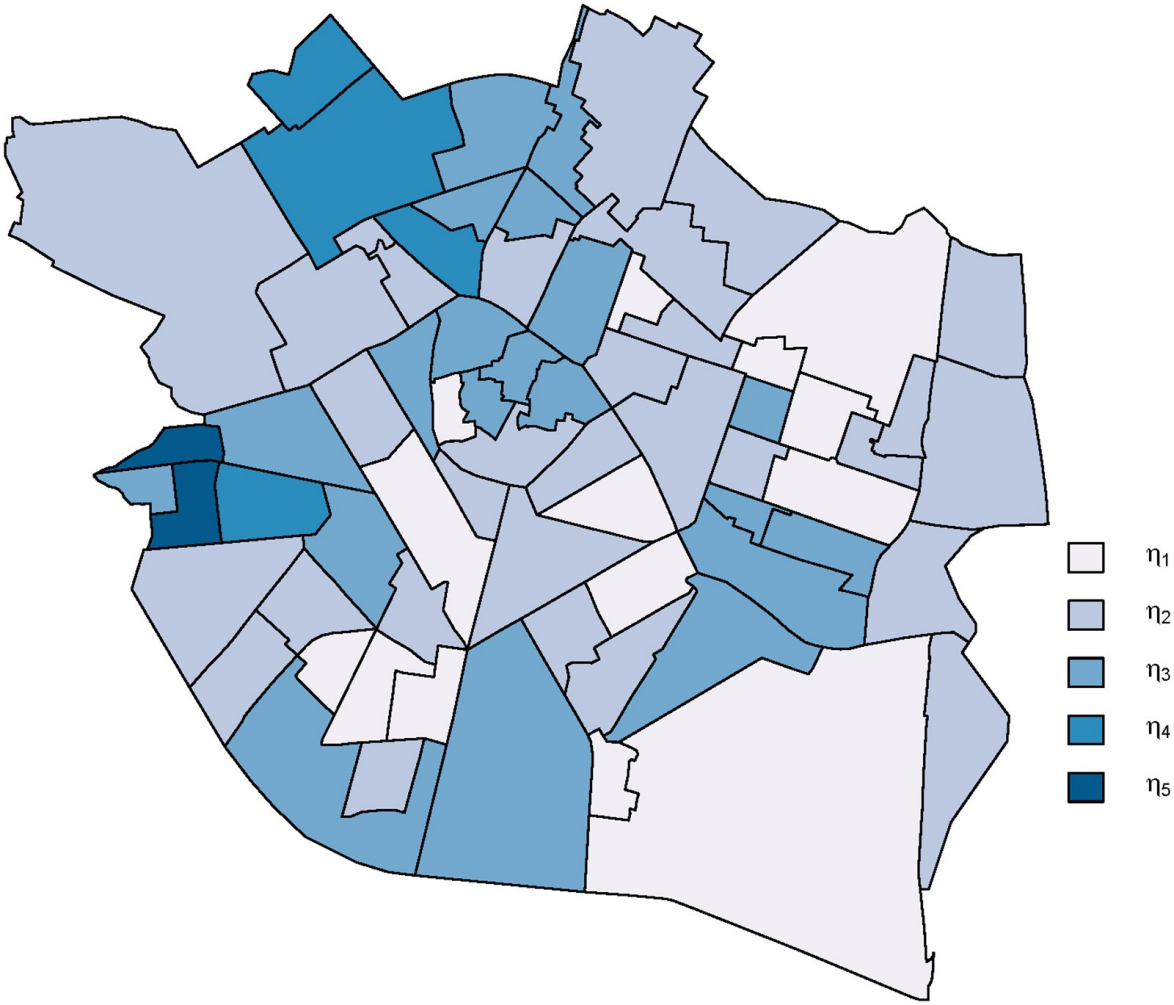
Case study: Clustering behavior of the varicella data for the 70 boroughs of the city of Valencia. The value of *k* = 5 has been selected as the number of risk classes.

The estimated posterior probabilities *f*(*z*_*i*_ = *E*_*j*_|*y*, *k* = 5, *e*), *E*_*j*_ being the *j*-th column of the identity matrix *I*_5_, allow us to assign each small area *i* to the risk class corresponding to the highest probability. Fig 1 maps the clustering behavior of the data, where each color corresponds to a risk level and areas represented with the same color indicate that they belong to the same risk class. Hence, geographic disease variation is described by a piecewise constant risk surface.

**Fig 2:**
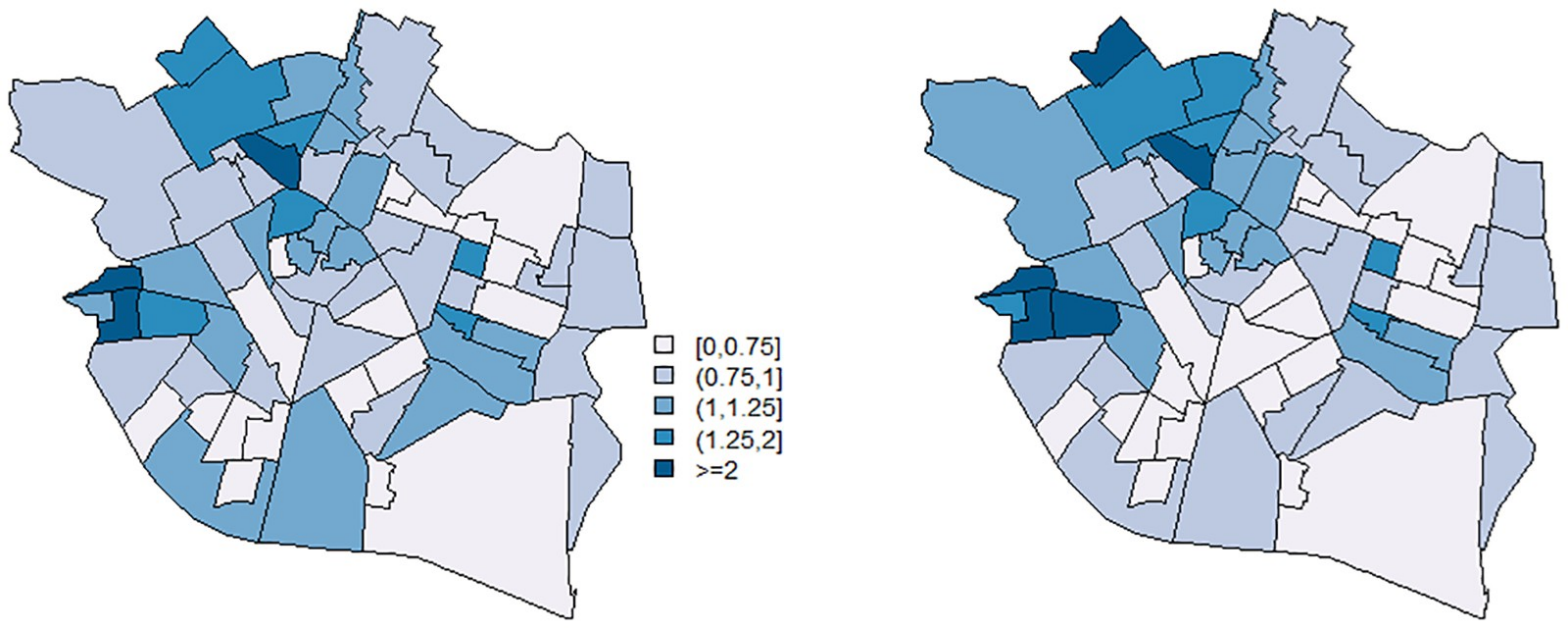
Case study: Estimated relative risks for the 70 boroughs of the city of Valencia. Left: Results obtained with our model. Right: Results obtained by applying the convolution model.

If we are interested in obtaining relative risk estimates for each borough, we can derive them using Eq (16), which allows us to incorporate model uncertainty. A summary of the estimated relative risks for the 70 boroughs of Valencia is shown in Table 8. Fig 2 maps the estimated relative risks. For comparative purposes, we also include the results obtained by the convolution model. Note that the estimated relative risks with our model are very similar to those provided by the convolution model. The log-pseudo marginal likelihood ($\sum_{i=1}^{m}log\left(CPO_{i}\right)$) corresponding to our model and that of the convolution model are, respectively, -178.30 and -213.41. So, the fit provided by the proposed model is slightly better than that of the convolution model. It is important to emphasize here that, even though our model does not impose any spatial correlation, it properly describes the spatial distribution of varicella in the city of Valencia.

**Table 8:** Case study: Summary of the estimated relative risks for the 70 boroughs of Valencia. The results obtained with the convolution model are also included.

	Min	Q1	Median	Mean	Q3	Max
Proposed model	0.46	0.74	0.89	1.06	1.06	5.34
Convolution model	0.49	0.67	0.89	1.06	1.11	5.62

**Fig 3:**
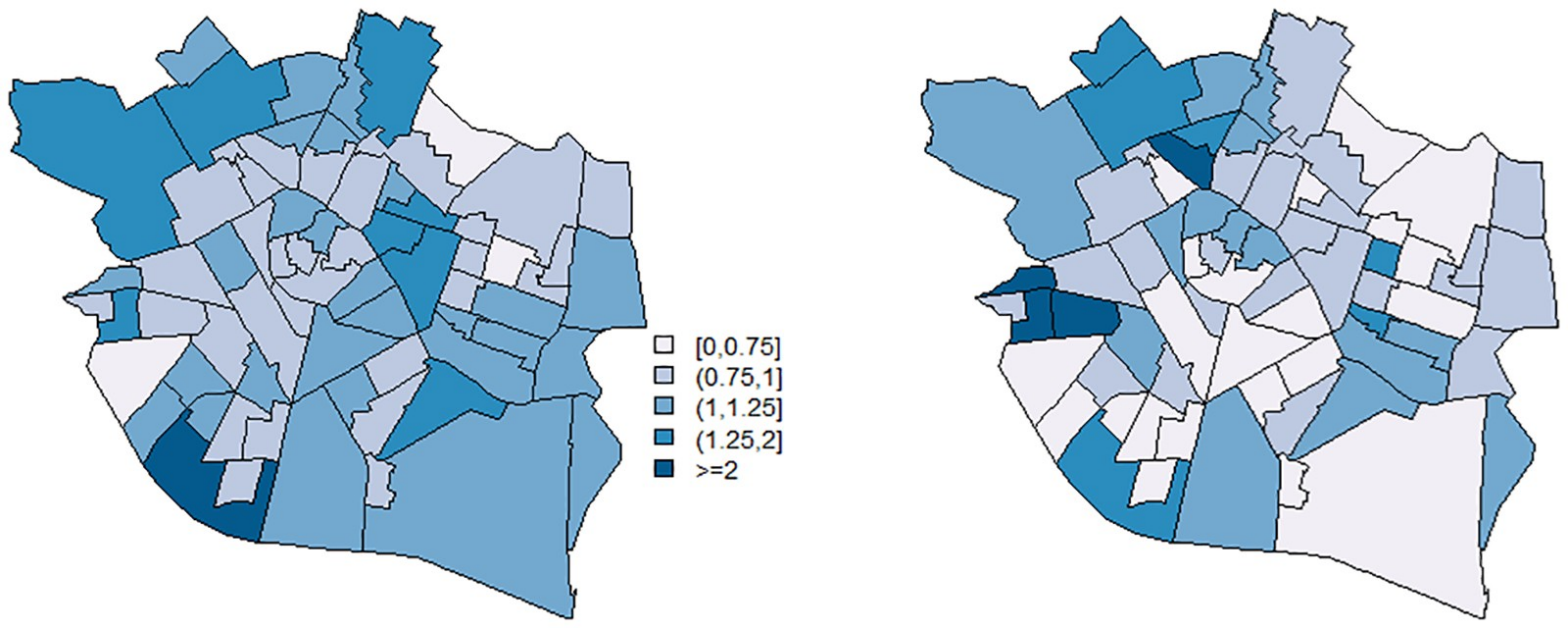
Case study. Left: Total impact of covariate. Right: Estimated mean of the Poisson distribution without the term *e*_*i*_ for the 70 boroughs of the city of Valencia.

We now show the results obtained in the analysis of the data when we include one covariate. In particular, for each borough, we have information corresponding to the percentage of population aged 0-4, which constitutes one of the high-risk age groups. Posterior point estimate of parameter *β* is 0.19, being the 95% confidence interval equal to [0.05, 0.31]. This estimate is consistent with the estimate obtained after fitting a model including the covariate effect and uncorrelated random effects (namely, *log*(*θ*_*i*_) = *α* + *βx*_*i*_ + *v*_*i*_, $v_{i}∼N\left(0,\sigma_{v}^{2}\right)$): $\hat{\beta }=0.15$, and *CI*_95%_(*β*) = [−0.007, 0.309]. From 2006 to 2013, varicella vaccine was available in pharmacies and young children could be vaccinated according to parents’ criteria. A more informative covariate would be the percentage of unvaccinated population aged 0-4. However, this information is not available at the borough level. Nevertheless, the proposed model is capable of estimating the effect of the available covariate and the residual relative risks for each borough. Fig 3 maps the total impact of covariate (*E*(exp{*βx*_*i*_}|*y*, *e*, *x*)) and the mean of the Poisson distribution without the term *e*_*i*_ (that is, $E(\text{exp}\left\{\beta x_{i}\right\}\cdot z_{i}^{\prime }\eta |y,e,x)$). As expected, for each borough, the estimated mean of the Poisson distribution is practically the same as the relative risks estimated with the proposed model without covariate information. The corresponding log-pseudo marginal likelihood is -176.45.

## Discussion

We have proposed a Bayesian Poisson mixture model that allows for relative risk estimation and cluster detection. By looking at the posterior distribution of each indicator vector *z*_*i*_, the small areas can be grouped together in, possibly multiple, risk classes. However, posterior averaging over both the generated samples and the number of risks classes considered leads to a smoothly varying risk surface.

Our model formulation allows that areas belonging to a specific risk class are disconnected. This idea has been explored in previous papers, but some kind of spatial dependence in the definition of the prior distributions for the allocation variables is always assumed [17, 18]. A major novelty of the proposed methodology is that we do not impose any spatial correlation at any level of the model hierarchy. However, as shown in the case study, the model can properly explain the spatial distribution of the data under study. The results obtained in a simulation study also demonstrate the good performance of our procedure in a variety of situations encompassing both smooth and discontinuous cases. This flexibility is of utter importance because, in practice, there is little prior information about the underlying risk surface.

It is also important to emphasize that we have treated the number of risks classes *k* as an additional parameter of the model. We have proposed here a novel methodology based on the marginal likelihood of the data given parameter *k* to estimate its posterior distribution. The integral to obtain such marginal distribution is solved by Monte Carlo integration using an approximation to the posterior distribution of the model parameters. As shown, this is an efficient and straightforward to program procedure.

If the effect of known explicative variables is taken into account, the proposed model aids in the identification of unexplained components of risk. Because our model formulation does not impose spatial correlation, it avoids the spatial confounding problem and provides a suitable framework to estimate the fixed-effect coefficients associated with spatially-structured covariates. Covariate information could also be used to introduce some spatial correlation in the model (for instance distance to putative foci of risk) or to define informative prior distributions for the indicator vectors. A very fruitful area for further research would be the incorporation and selection of multiple covariates.

It would also be valuable to extend the model to the spatio-temporal domain. This would allow us to describe the spatial distribution of disease risk and also its evolution over time. A possible extension can be obtained by defining, for each small area, a set of temporally correlated indicator vectors ${\left\{z_{it}\right\}}_{t=1}^{T}$. Because the assignment of each small area to a risk class can change, the model so defined would allow the relative risks to evolve in time.

## Supporting information

S1 Appendix Gibbs sampling procedure. (PDF)

S2 Appendix Identification of the number of risk classes: A comparison. (PDF)

S1 File (ZIP)
