# Supplementary material for: A Bayesian unified framework for risk estimation and cluster identification in small area health data analysis
Source: PLoS One. 2020 May 7;15(5):e0231935. doi: 10.1371/journal.pone.0231935 (PMC7205265; doi:10.1371/journal.pone.0231935)
Supplement: S1 Appendix — (PDF) [file pone.0231935.s001.pdf]

Supporting information

S1 Appendix. Gibbs sampling procedure.

In each iteration of the algorithm, we simulate a value for all the model parameters using the full conditional posterior distributions described in Section *MCMC algorithm*.

Sampling from $f(z_i = E_j|y, k, p, \eta, v, e)$ and $f(p|y, k, z, \eta, v, e)$ is straightforward. In order to sample from $f(\eta_j|y, k, z, p, \eta_{-j}, v, e)$, we have to deal with two situations depending on whether or not the j-th risk class is empty. In the first case, the full conditional posterior distribution corresponds to the prior distribution and we can use the inverse transform method. Let $x = \eta_j$, $A = v_{j-1}/(1 - v_{j-1})$, and $B = v_j/(1 - v_j)$. The cumulative distribution function of x is defined as:

$$F(x|A, B) = \int_A^x C\frac{1}{(1+u)^2}du = C((1+A)^{-1} - (1+x)^{-1}) = \frac{(1+A)^{-1} - (1+x)^{-1}}{(1+A)^{-1} - (1+B)^{-1}}$$

and the inverse of this cumulative distribution function is given by:

$$F^{-1}(y|A, B) = \left(\frac{1}{1+A} - \left(\frac{1}{1+A} - \frac{1}{1+B}\right)y\right)^{-1} - 1.$$

If the j-th risk class is not empty, let $x = \eta_j$, $a = y_{(j)} + 1$, $b = e_{(j)}$, and A and B defined as before. Since

$$Ga(x|a, b)\frac{1}{(1+x)^2} \leq Ga(x|a, b)\frac{1}{(1+A)^2}$$

when $A \leq x \leq B$, we can use an acceptance-rejection method to sample from the full conditional posterior distribution of each η_j. In particular, we propose to use the truncated Gamma in the interval $[A, B]$, that is $g(x) = Ga(x|a, b)$ if $A \leq x \leq B$, as the source density and the constant $M = 1/(1 + A)^2$ as the bound on $f(x)/g(x)$.

Finally, to sample from $f(v_j|y, k, z, p, \eta, v_{-j}, e)$, we can use the inverse transform method. Let $x = v_j$, $a = v_{j-1}$, $b = v_{j+1}$, $A = \frac{\eta_j}{1+\eta_j}$, $B = \frac{\eta_{j+1}}{1+\eta_{j+1}}$, and $\gamma_j = \gamma_{j+1} = 1$. The full conditional posterior distribution of $x = v_j$ can be written as:

$$f(x|a, b) = C\frac{1}{(x-a)(b-x)} \quad \text{if } A \leq x \leq B,$$

where the constant of proportionality C is equal to $(b - a)\left(log\frac{B-a}{b-B} - log\frac{A-a}{b-A}\right)^{-1}$. Note that this density is properly defined as $a \leq A \leq B \leq b$. The cumulative distribution function of x is then given by:

$$\begin{aligned} F(x) &= \int_A^x C\frac{1}{(u-a)(b-u)}du = C\int_A^x \frac{1}{b-a}\left(\frac{1}{u-a} + \frac{1}{b-u}\right)du \\ &= \frac{C}{b-a}\left(log\frac{x-a}{b-x} - log\frac{A-a}{b-A}\right), \end{aligned}$$

with inverse:

$$F^{-1}(y) = \frac{a + bk(y)}{1 + k(y)}, \quad \text{where} \quad k(y) = \frac{A-a}{b-A}\left(\frac{(B-a)(b-A)}{(b-B)(A-a)}\right)^y.$$
