# Supplementary material for: A Bayesian unified framework for risk estimation and cluster identification in small area health data analysis
Source: PLoS One. 2020 May 7;15(5):e0231935. doi: 10.1371/journal.pone.0231935 (PMC7205265; doi:10.1371/journal.pone.0231935)
Supplement: S2 Appendix — (PDF) [file pone.0231935.s002.pdf]

Supporting information

S2 Appendix. Identification of the number of risk classes: A comparison.

For comparative purposes, we show here the results that are obtained by applying the criterion proposed in [21] to identify the number of risk classes. For Scenarios 1 and 2, the mixture model was estimated assuming a relatively large number of risk classes, namely $K_{max} = 10$. The true number of latent classes was then estimated as the posterior mode of the number of non-empty classes, where a class is defined as empty if the proportion of small areas assigned to that risk class in a specific MCMC iteration is below a certain cut-off ψ. The values of ψ that we have considered are 0.1, 0.02, and 0.05. As a prior for the class proportions p we assumed the Dirichlet distribution $p \sim Dir(p|\alpha)$ with $\alpha_j = 2, \forall j$. However, as the authors conclude after an extensive simulation study, the criterion seems to yield the best results if the class proportions are given a symmetric Dirichlet prior with α slightly lower than $d/2$, d being the number of class-specific parameters. In the proposed model, the class-specific parameter is the latent risk η_j, so $d = 1$. Hence, we also considered the value $\alpha_j = 0.4, \forall j$.

For Scenario 1, Table 1 shows the percentage of data sets that select each possible value of k from 1 to $K_{max} = 10$ as the number of risk classes. As can be seen, the number of classes is overestimated when $\alpha_j = 2$, and the cut-off value has to be set equal to 0.1 so that the value of k chosen is closer to the real one. No such overestimation is observed if $\alpha_j = 0.4$, even if the value of k most often selected tends to be greater than the true value unless $\psi = 0.1$. The percentage of correspondence for $\alpha_j = 0.4$ and $\psi = 0.1$ is 91%.

Table 1. Scenario 1: Percentage of data sets that select each possible value of k from 1 to $K_{max} = 10$ as the number of risk classes. True value is $k = 3$.

k	1	2	3	4	5	6	7	8	9	10
$\psi = 0.1$, $\alpha = 2$	0%	0%	17%	83%	0%	0%	0%	0%	0%	0%
$\psi = 0.05$, $\alpha = 2$	0%	0%	0%	0%	7%	93%	0%	0%	0%	0%
$\psi = 0.02$, $\alpha = 2$	0%	0%	0%	0%	0%	0%	0%	96%	4%	0%
$\psi = 0.1$, $\alpha = 0.4$	0%	7%	91%	2%	0%	0%	0%	0%	0%	0%
$\psi = 0.05$, $\alpha = 0.4$	0%	0%	21%	78%	1%	0%	0%	0%	0%	0%
$\psi = 0.02$, $\alpha = 0.4$	0%	0%	0%	8%	91%	1%	0%	0%	0%	0%

Table 2 corresponds to Scenario 2. The most satisfactory results are obtained for the value $\alpha_j = 0.4$ and the cut-off $\psi = 0.02$. The corresponding percentage of correspondence achieved is 66%.

Table 2. Scenario 2: Percentage of data sets that select each possible value of k from 1 to $K_{max} = 10$ as the number of risk classes. True value is $k = 7$.

k	1	2	3	4	5	6	7	8	9	10
$\psi = 0.1$, $\alpha = 2$	0%	0%	0%	27%	73%	0%	0%	0%	0%	0%
$\psi = 0.05$, $\alpha = 2$	0%	0%	0%	0%	0%	0%	46%	54%	0%	0%
$\psi = 0.02$, $\alpha = 2$	0%	0%	0%	0%	0%	0%	0%	0%	67%	33%
$\psi = 0.1$, $\alpha = 0.4$	0%	0%	0%	32%	65%	3%	0%	0%	0%	0%
$\psi = 0.05$, $\alpha = 0.4$	0%	0%	0%	0%	2%	83%	15%	0%	0%	0%
$\psi = 0.02$, $\alpha = 0.4$	0%	0%	0%	0%	0%	0%	66%	34%	0%	0%
